# Several Lipid-Related Gene Polymorphisms Interact with Overweight/Obesity to Modulate Blood Pressure Levels

**DOI:** 10.3390/ijms130912062

**Published:** 2012-09-24

**Authors:** Rui-Xing Yin, Dong-Feng Wu, Lynn Htet Htet Aung, Ting-Ting Yan, Xiao-Li Cao, Xing-Jiang Long, Lin Miao, Wan-Ying Liu, Lin Zhang, Meng Li

**Affiliations:** 1 Department of Cardiology, Institute of Cardiovascular Diseases, The First Affiliated Hospital, Guangxi Medical University, Nanning 530021, Guangxi, China; E-Mails: wulove26@tom.com (D.-F.W.); lifeinnivana@hotmail.com (L.H.H.A); yan083199@163.com (T.-T.Y.); ma101996@gmail.com (X.-L.C.); longxingjiang2012@163.com (X.-J.L.); 2 Department of Cardiology, The Fourth Affiliated Hospital, Guangxi Medical University, Liuzhou 545005, Guangxi, China; E-Mail: caotianshuangmu@163.com; 3 Department of Cardiology, The Third Affiliated Hospital, Guangxi Medical University, Nanning 530000, Guangxi, China; E-Mail: liuwanying1224@yahoo.com.cn; 4 Department of Cardiology, The People’s Hospital of Guilin, Guilin 541001, Guangxi, China; E-Mail: bugemiulin@sina.com; 5 Department of Internal Medicine, Worker’s Hospital of Guangxi Liuzhou Iron and Steel (Group) Company, Liuzhou 545002, Guangxi, China; E-Mail: yimongxi@yahoo.com.cn

**Keywords:** blood pressure, hypertension, genetic polymorphism, overweight, obesity, interaction

## Abstract

Little is known about the interactions of single nucleotide polymorphisms (SNPs) and overweight/obesity on blood pressure levels. The present study was undertaken to detect 10 lipid-related gene SNPs and their interactions with overweight/obesity on blood pressure levels. Genotyping of ATP-binding cassette transporter A1 (ABCA-1) V825I, acyl-CoA:cholesterol acyltransferase-1 (ACAT-1) rs1044925, low density lipoprotein receptor (LDL-R) *Ava*II hepatic lipase gene (LIPC) −250G > A, endothelial lipase gene (LIPG) 584C > T, methylenetetrahydrofolate reductase (MTHFR) 677C > T, the E3 ubiquitin ligase myosin regulatory light chain-interacting protein (MYLIP) rs3757354, proprotein convertase subtilisin-like kexin type 9 (PCSK9) E670G, peroxisome proliferator-activated receptor delta (PPARD) +294T > C, and Scavenger receptor class B type 1 (SCARB1) rs5888 was performed in 978 normal weight and 751 overweight/obese subjects. The interactions were detected by factorial regression analysis. The genotypes of ACAT-1 AC, LIPC GA and AA, and SCARB1 TT; LDL-R A-A- and LIPC GA; and SCARB1 TT were interacted with overweight/obesity to increase systolic, diastolic blood pressure (SBP, DBP) and pulse pressure (PP) levels; respectively. The genotypes of ACAT-1 CC; ACAT-1 AA and CC were interacted with overweight/obesity to decrease SBP, PP levels (*p* < 0.01–0.001); respectively. The differences in blood pressure levels between normal weight and overweight/obese subjects might partly result from different interactions of several SNPs and overweight/obesity.

## 1. Introduction

Hypertension is an emerging risk factor that causes more than 7.1 million premature deaths a year worldwide [1], and that is becoming more prevalent in developing nations [2]. Hypertension can lead to coronary heart disease (CHD), stroke, congestive heart failure, renal insufficiency, and peripheral vascular disease. It is well known that blood pressure levels are regulated by multiple environmental and genetic factors and their interactions [3–6]. Recent genome-wide association studies in different populations have explored more than 160 candidate genes associated with blood pressure and hypertension, but the results of these association studies conducted with blood pressure traits are inconsistent [7–10]. A major reason for inconsistency among these studies may be different environmental modifiers that interact with genes to influence blood pressure and hypertension.

Obesity, the presence of excess body fat, has been clearly associated with cardiovascular disease, type 2 diabetes mellitus, gallbladder disease, cancers at several sites, osteoarthritis, and total mortality [11]. The prevalence of obesity has dramatically increased during recent years in all parts of the world [12]. According to the World Health Organization (WHO), more than 400 million adults were obese in 2005, and it is estimated that more than 700 million adults will be obese by 2015 [13]. Moreover, the rates of increase and the overall prevalence of obesity vary greatly across ethnic groups [14]. Among Americans, data from the National Health and Nutrition Examination Survey (NHANES) conducted in 2007–2008 showed that adults of 32.8% of non-Hispanic whites, 44.1% of non-Hispanic blacks, and 39.3% of Mexican-Americans were either overweight or obese [15]. The prevalence of overweight and obesity in Chinese was 24.1% and 2.8% in men and 26.1% and 5.0% in women; respectively [16]. Obesity has become a major clinical and public health problem that threatens to overwhelm already extended healthcare services in many countries. The link between overweight/obesity and blood pressure and hypertension has been well documented [17–26]. However, the interactions of single nucleotide polymorphisms (SNPs) and overweight/obesity on blood pressure levels are limited.

There are 56 ethnic groups in China. Han nationality is the largest ethnic group, and Yao nationality is the eleventh largest minority among the 55 minority groups according to the population size. Bai Ku Yao (White-trouser Yao), an isolated subgroup of the Yao minority, is named so because all men wear white knee-length knickerbockers. The population size is about 30,000. Because of isolation from the other ethnic groups, the special customs and cultures including their clothing, intra-ethnic marriages, dietary patterns, and corn wine and rum intakes are still completely preserved to the present day. Thus, Bai Ku Yao is thought to share the same ethnic ancestry and to possess a homogeneous genetic background, and is a useful subgroup for population genetic studies. In several previous epidemiological studies, we found that blood pressure levels and the prevalence of hypertension were lower in normal weight than in overweight/obese subjects [3,4]. We hypothesized that the differences in blood pressure levels between normal weight and overweight/obese subjects might partly result from different interactions of some SNPs and overweight/obesity in this population. Therefore, the aim of the present study was to detect 10 SNPs in different lipid-related genes and evaluate their interactions with overweight/obesity on blood pressure levels in the Guangxi Bai Ku Yao population. The SNPs were selected according to the previous findings of genome-wide association studies [7–10] and bioinformatics functional assessment. Computational analysis of 10 SNPs ascribed potential functional characteristics to each variant allele [27]. In addition, the 10 SNPs selected for genotyping also based on the frequency of Beijing Han population from the Human Genome Project Database. The heterozygosity values were higher than 10% for the minor allele frequency.

## 2. Results

### 2.1. General Characteristics

Table 1 shows the general characteristics and blood pressure levels of the participants. The levels of education, weight, body mass index (BMI), waist circumference, systolic blood pressure (SBP), diastolic blood pressure (DBP), serum total cholesterol (TC), triglyceride (TG), low-density lipoprotein cholesterol (LDL-C), apolipoprotein (Apo) A1, ApoB, and the percentages of subjects who consumed alcohol were higher in overweight/obese than in normal weight subjects (*p* < 0.05–0.001), whereas the levels of serum high-density lipoprotein cholesterol (HDL-C), the ratio of ApoA1 to ApoB, and the percentages of subjects who smoked cigarettes were lower in overweight/obese than in normal weight subjects (*p* < 0.01 for all). There were no significant differences in the levels of mean age, height, pulse pressure (PP), and the ratio of male to female between the overweight/obese and normal weight subjects (*p* > 0.05 for all).

### 2.2. Electrophoresis and Genotypes

**Figure 1:**
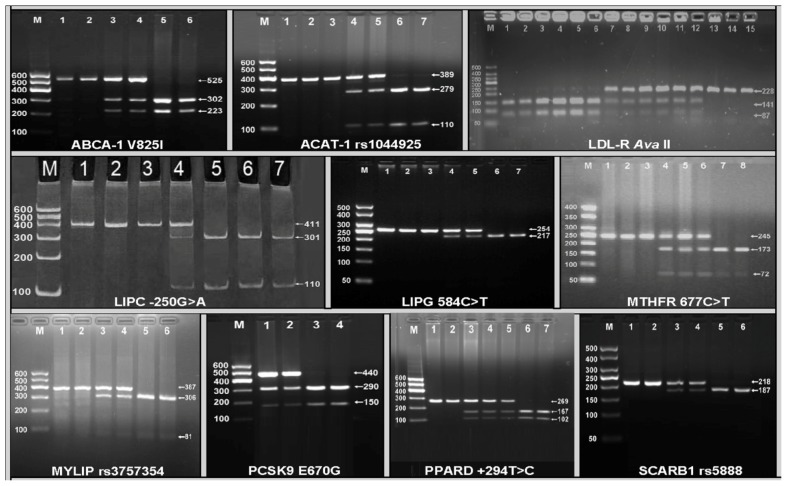
Genotyping of 10 lipid-related gene polymorphisms by polymerase chain reaction and restriction fragment length polymorphism (PCR-RFLP). **ABCA1**, ATP-binding cassette transporter A1. Lane M, 100 bp marker ladder; lanes 1 and 2, GG genotype (525 bp); lanes 3 and 4, GA genotype (525-, 302- and 223-bp); and lanes 5 and 6, AA genotype (302- and 223-bp); **ACAT-1**, acyl-CoA:cholesterol acyltransferase-1. Lane M, 100 bp marker ladder; lanes 1–3, AA genotype (389 bp); lanes 4 and 5, AC genotype (389-, 279- and 110-bp); and lanes 6 and 7, CC genotype (279- and 110-bp); **LDL-R**, low density lipoprotein receptor. Lane M, 50 bp marker ladder; lanes 1–6, A+A+ genotype (141- and 87-bp); lanes 7–12, A-A+ genotype (228-, 141- and 87-bp); and lanes 13–15, A-A- genotype (228-bp); **LIPC**, hepatic lipase gene. Lane M, 100 bp marker ladder; lanes 1–3, GG genotype (411 bp); lane 4, GA genotype (411-, 301- and 110-bp); and lanes 5–7, AA genotype (301- and 110-bp); **LIPG**, endothelial lipase gene. Lane M, 50 bp marker ladder; lane 1, the PCR product of the sample (254 bp); lanes 2 and 3, CC genotype (254 bp); lanes 4 and 5, CT genotype (254-, 217- and 37-bp); and lanes 6 and 7, TT genotype (217- and 37-bp). The 37 bp fragment was invisible in the gel owing to its fast migration speed; **MTHFR**, methylenetetrahydrofolate reductase. Lane M, 50 bp marker ladder; lanes 1–3, CC genotype (245 bp); lanes 4–6, CT genotype (245-, 173- and 72-bp); and lanes 7 and 8, TT genotype (173- and 72-bp); **MYLIP**, the E3 ubiquitin ligase myosin regulatory light chain-interacting protein. Lane M, 100 bp marker ladder; lanes 1 and 2, AA genotype (387 bp); lanes 3 and 4, AG genotype (387-, 306- and 81-bp); and lanes 5 and 6, GG genotype (306- and 81-bp); **PCSK9**, proprotein convertase subtilisin-like kexin type 9. Lane M, 100 bp marker ladder; lanes 1 and 2, PCR products of the samples (440 bp); lanes 3 and 4, AG genotype (440-, 290- and 150-bp); and lanes 5 and 6, AA genotype (290- and 150-bp). The GG homozygous of the PCSK9 E670G was not detected in our study population; **PPARD**, peroxisome proliferator-activated receptor delta. Lane M, 100 bp marker ladder; lanes 1 and 2, TT genotype (269 bp); lanes 3–5, TC genotype (269-, 167- and 102-bp); and lanes 6 and 7, CC genotype (167- and 102-bp); **SCARB1**, Scavenger receptor class B type 1. Lane M, 50 bp marker ladder; lanes 1 and 2, TT genotype (218 bp); lanes 3 and 4, CT genotype (218-, 187- and 31-bp); and lanes 5 and 6, CC genotype (187- and 31-bp). The 31 bp fragment was invisible in the gel owing to its fast migration speed.

The polymerase chain reaction (PCR) products of ATP-binding cassette transporter A1 (ABCA-1) V825I (rs2066715), acyl-CoA:cholesterol acyltransferase-1 (ACAT-1) rs1044925, low density lipoprotein receptor (LDL-R) *Ava*II, hepatic lipase gene (LIPC) −250G > A (rs2070895), endothelial lipase gene (LIPG) 584C > T (rs2000813), methylenetetrahydrofolate reductase (MTHFR) 677C > T (rs1801133), the E3 ubiquitin ligase myosin regulatory light chain-interacting protein (MYLIP, also known as IDOL) rs3757354, proprotein convertase subtilisin-like kexin type 9 (PCSK9) E670G (rs505151), peroxisome proliferator-activated receptor delta (PPARD) +294T > C (rs2016520) and Scavenger receptor class B type 1 (SCARB1) rs5888 were 525-, 389-, 228-, 411-, 254-, 254-, 387-, 440-, 269- and 218-bp nucleotide sequences; respectively. The genotypes of the 10 SNPs were shown in Figure 1.

### 2.3. Nucleotide Sequences

**Figure 2:**
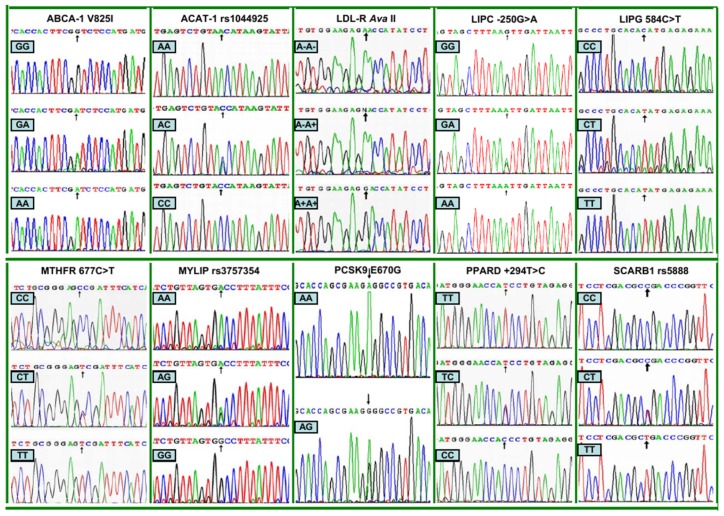
The parts of the nucleotide sequence of 10 lipid-related gene polymorphisms by direct sequencing. **ABCA-1**, ATP-binding cassette transporter A1; **ACAT-1**, acyl-CoA:cholesterol acyltransferase-1; **LDL-R**, low density lipoprotein receptor; **LIPC**, hepatic lipase gene; **LIPG**, endothelial lipase gene; **MTHFR**, methylenetetrahydrofolate reductase; **MYLIP**, the E3 ubiquitin ligase myosin regulatory light chain-interacting protein. **PCSK9**, proprotein convertase subtilisin-like kexin type 9; **PPARD**, peroxisome proliferator-activated receptor delta; **SCARB1**, Scavenger receptor class B type 1.

The genotypes detected by PCR-RFLP were also confirmed by direct sequencing (Figure 2).

### 2.4. Genotypic and Allelic Frequencies

The genotypic and allelic frequencies of the SNPs between normal weight and overweight/obese subjects are summarized in Table 2. The genotypic distribution of 10 SNPs fitted the Hardy-Weinberg equilibrium (*p* > 0.05 for all). The genotypic and allelic frequencies of LIPC and PCSK9 were different between normal weight and overweight/obese subjects, the overweight/obese subjects had higher LIPC −250A and PCSK9 670A allele frequencies than normal weight subjects (*p* < 0.05–0.001). The genotypic frequency of LIPG and allelic frequency of MYLIP were also different between normal weight and overweight/obese subjects (*p* < 0.05 for each). There were no significant differences in the genotypic and allelic frequencies of the remaining SNPs between normal weight and overweight/obese subjects (*p* > 0.05 for all). The GG homozygous of the PCSK9 E670G was not detected in our study population.

### 2.5. Genotypes and Blood Pressure Levels

**Figure 3:**
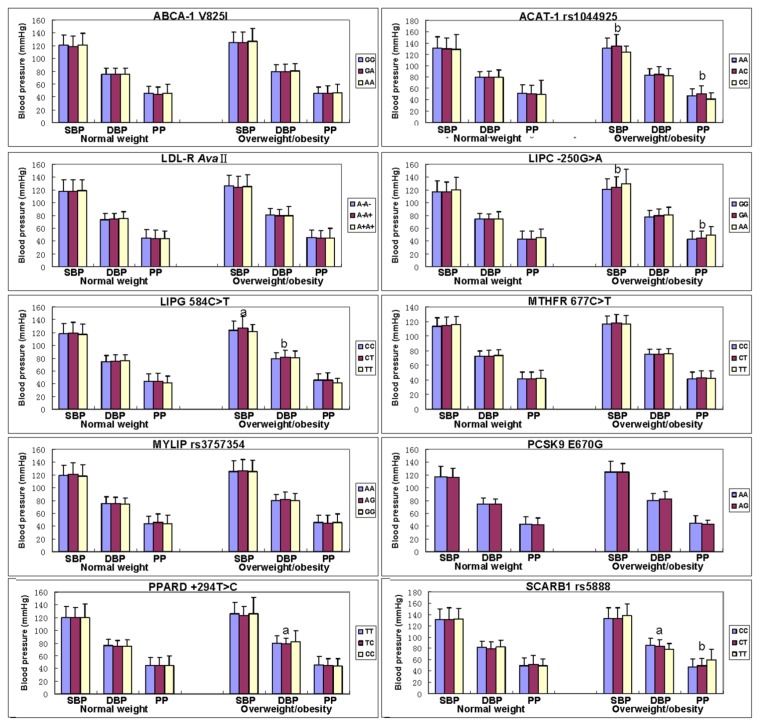
The genotypes of 10 SNPs and blood pressure levels between the normal weight and overweight/obese subjects. SBP, systolic blood pressure; DBP, diastolic blood pressure; PP, pulse pressure; ABCA-1, ATP-binding cassette transporter A1; ACAT-1, acyl-CoA:cholesterol acyltransferase-1; LDL-R, low density lipoprotein receptor; LIPC, hepatic lipase gene; LIPG, endothelial lipase gene; MTHFR, methylenetetrahydrofolate reductase; MYLIP, the E3 ubiquitin ligase myosin regulatory light chain-interacting protein; PCSK9, proprotein convertase subtilisin-like kexin type 9; PPARD, peroxisome proliferator-activated receptor delta; SCARB1, Scavenger receptor class B type 1. Sex, age, education level, physical activity, alcohol consumption, cigarette smoking, and hyperlipidemia have been adjusted for the statistical analysis. ^a^ *p* < 0.01 and ^b^ *p* < 0.001 (after permutation correction).

The association of genotypes and blood pressure parameters between normal weight and overweight/obese subjects is shown in Figure 3. The levels of SBP, DBP and PP in normal weight subjects were not different among the genotypes of all SNPs (*p >* 0.05 for all).

The levels of SBP (ACAT-1, LIPC and LIPG), DBP (LIPG, PPARD and SCARB1), and PP (ACAT-1, LIPC and SCARB1) in overweight/obese subjects were different among the genotypes (*p* < 0.01–0.001).

### 2.6. Interactions of the SNPs and Overweight/Obesity on Blood Pressure Levels

The interactions of 10 SNPs and overweight/obesity on blood pressure levels are given in Table 3. The SNPs of ABCA-1 (SBP and PP), LDL-R (DBP), LIPC (SBP and DBP), and SCARB1 (PP) were shown interactions with overweight/obesity to influence blood pressure levels (*p* < 0.01–0.001). ACAT-1 AA genotype interacted with overweight/obesity to decrease PP, AC genotype interacted with overweight/obesity to increase SBP, and CC genotype interacted with overweight/obesity to decrease SBP and PP. LDL-R A-A- genotype interacted with overweight/obesity to increase DBP. LIPC GA genotype interacted with overweight/obesity to increase SBP and DBP, and AA genotype interacted with overweight/obesity to increase SBP. SCARB1 TT genotype interacted with overweight/obesity to increase PP.

### 2.7. Correlation between Genotypes or Alleles and Blood Pressure Parameters

The results of multiple linear regression analysis between genotypes or alleles and blood pressure parameters are shown in Table 4. Blood pressure levels were also associated with the genotypes or alleles of several SNPs in the combined population of normal weight and overweight/obese subjects, and overweight/obese subjects; respectively (*p* < 0.05–0.001).

## 3. Experimental Section

### 3.1. Study Population

A total of 1729 unrelated participants of Bai Ku Yao who reside in Lihu and Baxu villages in Nandan County, Guangxi, China were randomly selected from our previous stratified randomized cluster samples [3,4]. The age of the subjects ranged from 15 to 86 years, with an average age of 41.38 ± 14.71 years. There were 978 normal weight (490 males and 488 females) and 751 overweight/obese subjects (378 men and 373 women). All of the subjects were rural agricultural workers. The subjects had no evidence of diseases related to atherosclerosis, CHD and diabetes. None of them had been treated with antihypertensive drugs (such as nifedipine and/or captopril, beta-blockers, and diuretics), lipid-lowering drugs, hormones, or contraceptive drugs. The protocol was approved by the Ethics Committee of the First Affiliated Hospital, Guangxi Medical University. Informed consent was obtained from each participant.

### 3.2. Epidemiological Survey

The survey was carried out using internationally standardized methods [3,4]. Trained interviewers administered questionnaires to gather information on each participant’s demographic characteristics, socioeconomic status, lifestyle factors, and medical and medication history. Blood pressure was measured three times by a well-trained physician with the use of a standard mercury sphygmomanometer after the subjects had been seated for more than 5 min, and the average of three measurements was used for the analysis. SBP was determined by the first Korotkoff sound, and DBP by the fifth Korotkoff sound. PP was calculated as the SBP minus the DBP. Height was measured to the nearest 0.1 cm on a portable stadiometer. Weight was measured to the nearest 0.1 kg with the subjects standing motionless on the scale. BMI was calculated as weight in kilograms divided by the square of height in meters. Waist circumference was measured with a nonstretchable measuring tape, at the level of the smallest area of the waist, to the nearest 0.1 cm.

### 3.3. Biochemical Measurements

Fasting venous blood samples of 5 mL were obtained from all subjects. A part of the sample (2 mL) was collected into glass tubes and used to determine serum lipid levels. Another part of the sample (3 mL) was transferred to tubes with anticoagulate solution (4.80 g/L citric acid, 14.70 g/L glucose, and 13.20 g/L tri-sodium citrate) and used to extract deoxyribonucleic acid (DNA). The levels of TC, TG, HDL-C, and LDL-C in samples were determined by enzymatic methods. Serum ApoA1 and ApoB levels were detected by the immunoturbidimetric immunoassay. All determinations were performed with an autoanalyzer (Type 7170A; Hitachi Ltd., Tokyo, Japan) in our Clinical Science Experiment Center [3,4].

### 3.4. Genetic Analyses

Genomic DNA was extracted from the peripheral blood leukocytes by the phenol-chloroform method [5]. Genotyping of ABCA-1, ACAT-1, LDL-R, LIPC, LIPG, MTHFR, MYLIP, PCSK9, PPARD, and SCARB1 SNPs was performed using PCR-RFLP. The sequences of the forward and backward primers and restriction enzyme used for the genotyping of 10 SNPs, the thermocycling protocol, the approach to electrophoresis, and the procedures for quality control have been described previously [5]. Genotypes were scored by an experienced reader blinded to epidemiological data and blood pressure levels.

### 3.5. DNA Sequencing

Fifty-eight samples (each genotype in two; respectively) detected by the PCR-RFLP were also confirmed by direct sequencing. The PCR products were purified by low melting point gel electrophoresis and phenol extraction, and then the DNA sequences were analyzed in Shanghai Sangon Biological Engineering Technology & Services Co., Ltd., China.

### 3.6. Diagnostic Criteria

Hypertension was defined as an average SBP of 140 mmHg or greater and/or an average DBP of 90 mmHg or greater [3,4]. The normal values of serum TC, TG, HDL-C, LDL-C, ApoA1 and ApoB levels, and the ratio of ApoA1 to ApoB in our Clinical Science Experiment Center were 3.10–5.17, 0.56–1.70, 0.91–1.81, 2.70–3.20 mmol/L, 1.00–1.78, 0.63–1.14 g/L, and 1.00–2.50; respectively. The individuals with TC > 5.17 mmol/L and/or TG > 1.70 mmol/L were defined as hyperlipidemic [3,4]. The diagnostic criteria of overweight and obesity were according to the Cooperative Meta-analysis Group of China Obesity Task Force. Normal weight, overweight and obesity were defined as a BMI < 24, 24–28, and > 28 kg/m^2^; respectively [3,4].

### 3.7. Statistical Analysis

Data are presented as means ± SD for continuous variables and as frequencies or percentages for categorical variables. Chi square tests were used to compare the differences in percentages and to assess Hardy-Weinberg expectations. Differences in mean values were assessed using analysis of covariance (ANCOVA) and unpaired *t* tests. Potential confounding factors were sex, age, education level, physical activity, alcohol consumption, cigarette smoking, and hyperlipidemia. All significant associations were further corrected for multiple tests by a permutation test. The permutation test was conducted by changing the orders of dependent variable randomly against the genotypes (under the null hypothesis—no association between dependent variable and haplotypes). This process was repeated 1000 times. The *p* values of 1000 permutations were sorted in a descending manner. If the observed *p* value is less than or equal to the 950th *p* value, the association was considered statistically significant. The allelic and genotypic frequencies were calculated from the observed genotypic counts. The interactions of 10 SNPs and overweight/obesity on blood pressure levels were assessed by using a factorial regression analysis after controlling for potential confounders. Multiple linear regression was used to ascertain the correlation between genotypes (ABCA-1: GG = 1, GA = 2, AA = 3; ACAT-1: AA = 1, AC = 2, CC = 3; LDL-R: A−A− = 1, A−A+ = 2, A+A+ = 3; LIPC: GG = 1, GA = 2, AA = 3; LIPG: CC = 1, CT = 2, TT = 3; MTHFR: CC = 1, CT = 2, TT = 3; MYLIP: AA = 1, AG = 2, GG = 3; PCSK9: AA = 1, AG = 2; PPARD: TT = 1, TC = 2, CC = 3; and SCARB1: CC = 1, CT = 2, TT = 3) or alleles (the minor allele noncarrier = 1, the minor allele carrier = 2) and blood pressure parameters in the combined population of normal weight and overweight/obese subjects, normal weight subjects, and overweight/obese subjects; respectively.

## 4. Discussion

In the present study, we showed that the genotypic and allelic frequencies of LIPC and PCSK9 were different between normal weight and overweight/obese subjects, the overweight/obese subjects had higher LIPC-250A and PCSK9 670A allele frequencies than normal weight subjects. The genotypic frequency of LIPG and allelic frequency of MYLIP were also different between normal weight and overweight/obese subjects. These results indicate that several lipid-related gene SNPs may involve in the regulation of blood pressure. Both dyslipidemia and hypertension are the components of the metabolic syndrome. Blood pressure and serum lipid levels have been found to be consistently related in several previous studies [28,29]. Some studies have prospectively examined the relationship between serum lipid levels and the future development of hypertension, finding that there is an association between serum lipid levels and the development of hypertension [30,31]. Higher levels of serum TC, non-HDL-C, and the TC/HDL-C ratio were independently associated with a subsequent increased risk of incident hypertension in apparently healthy men. Elevated serum lipid levels appeared to predate the onset of hypertension by years [31,32]. In recent years, a number of studies such as genetic linkage analyses and/or genome-wide association studies have been performed to elucidate the contribution of genetic factors to both conditions [7–10,33,34]. These genetic observations indicate that multiple genetic factors exist that may affect both blood pressure and serum lipid levels. Some genes involved in lipid metabolism may be involved in the genetic component of the development of hypertension.

The relationship between obesity and hypertension is well recognized. Overweight and obesity increase the risk of elevated blood pressure. The prevalence of hypertension was 2- to 6-fold higher in obesity than in normal weight crowd [17–19]. An increase in BMI and a decrease in BMI were significantly associated with increased and decreased SBP and DBP, respectively, compared to a stable BMI in both genders and all age groups [17,18]. In the Nurses’ Health Study [19], women with a BMI of 32 or greater had approximately six times higher risk of developing hypertension compared with women whose BMIs were less than 23. The mechanisms for the relation between obesity and blood pressure have not been fully elucidated. Insulin resistance and peripheral hyperinsulinemia resulting from overweight and obesity may play a critical role in the development of hypertension [20,21]. Other postulated factors include excessive caloric intake, enhanced sympathetic activity [22], and even the greater lean mass present in obese subjects. In addition, enhanced salt sensitivity [19], activated renin-angiotensin-aldosterone system [23], potentiated procoagulatory activity [24], and induced endothelial dysfunction [25,26] among obese individuals may be also responsible for the development of hypertension.

Several previous studies have evaluated the association between several SNPs and blood pressure variation [35–49]. However, the findings are inconsistent. Liu *et al.* [35] found that there was no difference in the genotypic and allelic frequencies of LDL-R *Ava*II between normotensives and essential hypertensives. Rodríguez-Esparragón *et al*. [36] reported that TT genotype of MTHFR C677T was associated with an increased risk of hypertension in males. A meta-analysis showed that the MTHFR C677T was consistently associated with severe diastolic hypertension during pregnancy [37]. The MTHFR C667T modulated baseline DBP and DBP responsiveness by short-term treatment of benazapril [38,39]. A significant association between MTHFR C677T polymorphism and hypertension/hypertension-in-pregnancy in both Caucasian and Asian populations was also observed in a recent meta-analysis [40]. The patients carrying MTHFR 677T allele were at increased risk of hypertension. The frequency of co-occurrence of MTHFR 677CT/1298CC genotypes was significantly higher in patients compared to controls (*p* < 0.05) and was associated with increased risk of hypertension [41]. Marinho *et al.* [42] showed that genotype distributions of MTHFR differ significantly between control and hypertensives with a greater prevalence of CT genotype. The MTHFR 677C allele was significantly more frequent in controls compared with patients with essential hypertension (*p* < 0.05), and CC genotype was more frequent in controls compared to patients with essential hypertension [43]. However, several studies showed that there was no association of MTHFR C677T and the prevalence of hypertension or blood pressure levels [44–46]. Gao *et al*. [47] reported that PPAR-gamma2 is associated with hypertension in the Han population of Inner Mongolia. The frequency of Ala allele was lower in patients with hypertension (1.3%) than in controls (3.6%, *p* = 0.018). The Pro12Ala polymorphism in PPAR-gamma is associated with blood pressure in subjects with type 2 diabetes. The subjects with Pro/Ala (24%) or Ala/Ala (2%) had lower DBP when adjusted for age and gender compared with Pro/Pro subjects (74%). This association was restricted to men, who also had a borderline significant difference in SBP [48]. However, Yan *et al.* [49] reported that the frequencies of the PPARD +294T > C genotypes were not different among the groups of metabolic syndrome, essential hypertension, and type 2 diabetes mellitus. In the current study, we showed that the levels of SBP (ACAT-1, LIPC and LIPG), DBP (LIPG, PPARD and SCARB1), and PP (ACAT-1, LIPC and SCARB1) in overweight/obese subjects but not in normal weight participants were different among the genotypes. To the best of our knowledge, the association between these SNPs and blood pressure variation in overweight/obese subjects has not been previously reported.

The interactions of 10 SNPs and overweight/obesity on blood pressure levels are not fully known. In the present study, we showed that the genotypes of ACAT-1 AC, LIPC GA and AA, and SCARB1 TT interacted with overweight/obesity to increase SBP levels, whereas the genotype of ACAT-1 CC interacted with overweight/obesity to decrease SBP levels. The genotypes of LDL-R A-A- and LIPC GA interacted with overweight/obesity to increase DBP levels. The genotype of SCARB1 TT interacted with overweight/obesity to increase PP levels, whereas the genotypes of ACAT-1 AA and CC interacted with overweight/obesity to decrease PP levels. These findings suggest that some blood pressure parameters in our study subjects were partly influenced by the interactions of several lipid-related SNPs and overweight/obesity. Lose weight and other healthy lifestyles are necessary for maintaining of normal blood pressure. To the best of our knowledge, the interactions of these SNPs and overweight/obesity on blood pressure parameters have not been previously explored.

The present study has several potential limitations. First, the levels of education, weight, and the percentages of subjects who consumed alcohol were higher in overweight/obese than in normal weight subjects, whereas the percentages of subjects who smoked cigarettes were lower in overweight/obese than in normal weight subjects. Although sex, age, education level, physical activity, alcohol consumption, cigarette smoking, and hyperlipidemia have been adjusted for the statistical analysis, we could not completely eliminate the potential effects of these factors on blood pressure levels among different genotypes in both groups. Second, the diet was not adjusted for the statistical analysis. In the present study, the population of Bai Ku Yao is a special and isolated ethnic subgroup of the Yao minority in China. The special customs and cultures including their clothing, intra-ethnic marriages, diet and lifestyle are still completely conserved to the present day. The diet in this population is consistent throughout the year and among individuals because of the Bai Ku Yao’s reliance on a limited number of locally available food items. Their staple food is corn gruel or corn tortillas. On ordinary days, they are vegetarians [3,4]. Third, it is clearly established that blood pressure levels are regulated by multiple environmental and genetic factors, and their interactions. Although we have detected the interactions of 10 lipid-related gene SNPs and overweight/obesity on blood pressure levels in this study, there are still many unmeasured environmental and genetic factors and their interactions. Thus, the interactions of gene-gene, gene-environment, and environment-environment on blood pressure levels remain to be determined. Finally, Bai Ku Yao is an isolated subgroup of the Yao minority in China. No replication of the interactions between genetic polymorphisms and overweight/obesity on blood pressure variation was obtained in this independent population. Thus, it may not generalize findings in other populations worldwide.

## 5. Conclusions

Several lipid-related SNPs in overweight/obese subjects were found to be associated with blood pressure levels in the Guangxi Bai Ku Yao population. The interactions of ABCA-1 (SBP and PP), LDL-R (DBP), LIPC (SBP and DBP), and SCARB1 (PP) and overweight/obesity on blood pressure levels were also detected. The differences in blood pressure levels between normal weight and overweight/obese subjects might partly result from different interactions of several lipid-related gene SNPs and overweight/obesity. However, large studies of populations with different ethnic origins are required to confirm these observations.

## Figures and Tables

**Table 1:** The general characteristics and blood pressure levels between normal weight and overweight/obese subjects.

Characteristics	Normal weight	Overweight/obesity	*t* (χ^2^)	*p*
Number	978	751	-	-
Male/female	490/488	378/373	0.009	0.924
Age, years	41.48 ± 16.21	41.25 ± 12.50	0.332	0.740
Education level, years	3.71 ± 3.89	4.71 ± 4.49	−4.170	0.000
Height, cm	153.73 ± 7.57	154.29 ± 8.49	−1.461	0.144
Weight, kg	50.28 ± 6.20	63.19 ± 8.86	−34.042	0.000
Body mass index, kg/m^2^	21.23 ± 1.68	26.48 ± 2.59	−48.285	0.000
Waist circumference, cm	70.58 ± 6.56	82.90 ± 7.39	−30.766	0.000
Alcohol consumption, *n* (%)	373 (38.1)	362 (48.2)	23.034	0.000
Cigarette smoking, *n* (%)	305 (31.2)	178 (23.7)	15.036	0.001
Systolic blood pressure, mmHg	119.69 ± 17.40	125.84 ± 17.64	−7.242	0.000
Diastolic blood pressure, mmHg	75.11 ± 9.98	80.57 ± 11.13	−10.563	0.000
Pulse pressure, mmHg	44.60 ± 12.87	45.36 ± 12.11	−1.251	0.211
Total cholesterol, mmol/L	4.46 ± 0.94	5.01 ± 1.05	−11.325	0.000
Triglyceride, mmol/L	1.21 ± 1.02	1.74 ± 1.50	−8.837	0.000
HDL-C, mmol/L	1.80 ± 0.47	1.73 ± 0.41	3.099	0.002
LDL-C, mmol/L	2.52 ± 0.73	2.96 ± 0.85	−11.180	0.000
Apolipoprotein (Apo) A1, g/L	1.37 ± 0.31	1.40 ± 0.27	−2.009	0.045
ApoB, g/L	0.84 ± 0.22	0.98 ± 0.24	−12.466	0.000
ApoA1/ApoB	1.75 ± 0.70	1.53 ± 0.58	7.256	0.000

Values are means ± SD or number of subjects (%). HDL-C, high-density lipoprotein cholesterol; LDL-C, low-density lipoprotein cholesterol.

**Table 2:** The genotypic and allelic frequencies between the subjects with normal weight and overweight/obesity [*n* (%)].

SNP	Genotype (Allele)	Normal weight (*n* = 978)	Overweight/obesity (*n* = 751)	χ^2^	*p*
ABCA-1 V825I (rs2066715)	GG	326 (33.3)	269 (35.8)		
	GA	480 (49.1)	334 (44.5)		
	AA	172 (17.6)	148 (19.7)	3.708	0.157
	G	1132 (57.9)	872 (58.1)		
	A	824 (42.1)	630 (41.9)	0.012	0.914

ACAT-1 (rs1044925)	AA	662 (67.7)	527 (70.2)		
	AC	279 (28.5)	205 (27.3)		
	CC	37 (3.8)	19 (2.5)	2.671	0.263
	A	1603 (82.0)	1259 (83.8)		
	C	353 (18.0)	243 (16.2)	2.080	0.149

LDL-R *Ava*II	A−A−	527 (53.9)	389 (51.8)		
	A−A+	371 (37.9)	295 (39.3)		
	A+A+	80 (8.2)	67 (8.9)	0.824	0.662
	A−	1425 (72.9)	1073 (71.4)		
	A+	531 (27.1)	429 (28.6)	0.848	0.357

LIPC −250G > A (rs2070895)	GG	480 (49.1)	233 (31.0)		
	GA	425 (43.5)	432 (57.5)		
	AA	73 (7.5)	86 (11.5)	57.882	0.000
	G	1385 (70.8)	898 (59.8)		
	A	571 (29.2)	604 (40.2)	45.999	0.000

LIPG 584C > T (rs2000813)	CC	454 (46.4)	308 (41.0)		
	CT	477 (48.8)	412 (54.9)		
	TT	47 (4.8)	31 (4.1)	6.314	0.043
	C	1385 (70.8)	1028 (68.4)		
	T	571 (29.2)	474 (31.6)	2.255	0.133

MTHFR 677C > T (rs1801133)	CC	471 (48.2)	354 (47.1)		
	CT	441 (45.1)	341 (45.4)		
	TT	66 (6.7)	56 (7.5)	0.404	0.817
	C	1383 (70.7)	1049 (69.8)		
	T	573 (29.3)	453 (30.2)	0.305	0.581

MYLIP (rs3757354)	AA	230 (23.5)	148 (19.7)		
	AG	477 (48.8)	363 (48.3)		
	GG	271 (27.7)	240 (32.0)	5.431	0.066
	A	937 (47.9)	659 (43.9)		
	G	1019 (52.1)	843 (56.1)	5.550	0.018

PCSK9 E670G (rs505151)	AA	916 (93.7)	721 (96.0)		
	AG	62 (6.3)	30 (4.0)		
	GG	0	0	4.636	0.031
	A	1894 (96.8)	1472 (98.0)		
	G	62 (3.2)	30 (2.0)	4.509	0.034

PPARD +294T > C (rs2016520)	TT	559 (57.2)	396 (52.7)		
	TC	354 (36.2)	312 (41.5)		
	CC	65 (6.6)	43 (5.7)	5.239	0.073
	T	1472 (75.3)	1104 (73.5)		
	C	484 (24.7)	398 (26.5)	1.375	0.241

SCARB1 (rs5888)	CC	548 (56.0)	417 (55.5)		
	CT	390 (39.9)	311 (41.4)		
	TT	40 (4.1)	23 (3.1)	1.497	0.473
	C	1486 (76.0)	1145 (76.2)		
	T	470 (24.0)	357 (23.8)	0.032	0.859

Values are number of subjects (%). SNP, single nucleotide polymorphism; ABCA-1, ATP-binding cassette transporter A1; ACAT-1, acyl-CoA:cholesterol acyltransferase-1; LDL-R, low density lipoprotein receptor; LIPC, hepatic lipase gene; LIPG, endothelial lipase gene; MTHFR, methylenetetrahydrofolate reductase; MYLIP, the E3 ubiquitin ligase myosin regulatory light chain-interacting protein; PCSK9, proprotein convertase subtilisin-like kexin type 9; PPARD, peroxisome proliferator-activated receptor delta; SCARB1, Scavenger receptor class B type 1.

**Table 3:** Interactions of several lipid-related gene polymorphisms and overweight/obesity on blood pressure levels.

SNP	Genotype	Normal weight			Overweight/obesity		
	
		SBP	DBP	PP	SBP	DBP	PP
ABCA-1 V825I (rs2066715)	GG	120.5 ± 15.9	75.4 ± 10.0	45.2 ± 11.2	125.5 ± 15.4	80.0 ± 10.1	45.6 ± 10.2
	GA	118.8 ± 16.3	75.0 ± 9.6	43.8 ± 11.9	125.1 ± 16.5	79.6 ± 11.3	45.5 ± 11.4
	AA	120.9 ± 18.6	75.5 ± 9.2	45.5 ± 14.3	126.5 ± 20.1	80.1 ± 12.2	46.4 ± 13.4
	*F*	2.127	0.262	2.309	0.199	0.079	0.724
	*p*	0.120	0.770	0.100	0.820	0.924	0.485

ACAT-1 (rs1044925)	AA	130.6 ± 20.2	79.4 ± 10.6	51.2 ± 15.0	130.3 ± 18.3	83.5 ± 11.3	46.8 ± 12.5↓
	AC	129.6 ± 19.0	79.7 ± 11.1	49.9 ± 15.3	135.1 ± 20.1↑	84.9 ± 13.9	50.2 ± 13.9
	CC	129.0 ± 25.5	79.8 ± 12.7	49.2 ± 24.9	123.5 ± 11.5↓	82.1 ± 12.7	41.5 ± 10.4↓
	*F*	0.338	0.112	1.048	5.825	1.289	5.910
	*p*	0.714	0.894	0.351	0.000 ^c^	0.276	0.000 ^c^

LDL-R *Ava*II	A−A−	118.0 ± 17.5	73.4 ± 9.8	44.5 ± 13.2	126.3 ± 16.7	80.5 ± 10.8↑	45.8 ± 11.7
	A−A+	118.1 ± 18.0	74.2 ± 9.3	43.9 ± 12.9	123.9 ± 17.3	79.2 ± 10.0	44.7 ± 11.4
	A+A+	118.9 ± 17.1	74.9 ± 10.5	44.0 ± 11.2	124.7 ± 18.9	79.8 ± 13.7	45.0 ± 15.2
	*F*	0.142	1.420	0.340	2.249	4.276	0.538
	*p*	0.867	0.242	0.712	0.106	0.003 ^c^	0.548

LIPC −250G > A (rs2070895)	GG	117.1 ± 16.9	74.2 ± 9.3	42.9 ± 12.7	120.6 ± 16.9	77.8 ± 9.7	42.8 ± 13.1
	GA	116.8 ± 15.8	73.8 ± 8.9	43.0 ± 12.3	123.8 ± 16.4↑	79.5 ± 10.4↑	44.3 ± 10.9
	AA	119.8 ± 19.2	74.3 ± 11.8	45.5 ± 13.5	129.7 ± 22.0↑	80.2 ± 12.6	49.5 ± 13.1
	*F*	1.347	0.299	1.685	4.233	3.125	2.367
	*p*	0.261	0.741	0.186	0.003^c^	0.009 ^c^	0.094

LIPG 584C > T (rs2000813)	CC	117.8 ± 15.8	74.4 ± 9.5	43.37 ± 12.30	123.6 ± 13.9	78.2 ± 10.5	45.3 ± 10.5
	CT	119.1 ± 17.0	75.3 ± 9.6	43.81 ± 12.60	126.8 ± 18.2	81.1 ± 11.0	45.7 ± 11.6
	TT	116.8 ± 16.2	76.0 ± 8.8	41.07 ± 10.72	121.5 ± 11.1	80.1 ± 10.9	41.4 ± 6.8
	*F*	1.214	1.452	1.261	0.920	2.408	0.087
	*p*	0.297	0.235	0.284	0.399	0.090	0.917

MTHFR 677C > T (rs1801133)	CC	113.7 ± 11.7	72.6 ± 7.5	41.1 ± 9.7	116.4 ± 10.9	74.8 ± 7.5	41.7 ± 9.3
	CT	114.1 ± 11.9	73.0 ± 7.3	41.1 ± 9.5	118.1 ± 11.7	75.2 ± 6.6	43.0 ± 9.6
	TT	115.8 ± 10.5	73.4 ± 7.6	42.5 ± 10.4	116.8 ± 11.7	75.7 ± 7.6	42.6 ± 10.2
	*F*	1.057	0.604	0.660	1.312	0.005	1.186
	*p*	0.348	0.547	0.517	0.270	0.995	0.306

MYLIP (rs3757354)	AA	118.9 ± 16.3	75.5 ± 9.9	43.5 ± 11.8	125.5 ± 16.5	79.5 ± 9.9	46.0 ± 11.2
	AG	120.8 ± 17.7	75.4 ± 10.0	45.4 ± 13.3	126.3 ± 17.9	81.5 ± 11.2	44.8 ± 12.0
	GG	118.4 ± 17.6	74.4 ± 10.0	44.1 ± 12.9	125.4 ± 18.0	79.9 ± 11.6	45.8 ± 12.8
	*F*	2.122	1.194	2.224	0.293	1.648	2.565
	*p*	0.120	0.303	0.109	0.746	0.193	0.077

PCSK9 E670G (rs505151)	AA	117.2 ± 16.3	74.2 ± 9.6	42.9 ± 12.0	124.2 ± 16.9	79.8 ± 11.0	44.4 ± 12.1
	AG	116.0 ± 15.0	74.1 ± 8.1	41.8 ± 11.3	124.7 ± 13.1	82.2 ± 11.9	42.5 ± 6.4
	*F*	0.418	0.006	0.620	0.457	1.868	0.085
	*p*	0.518	0.938	0.431	0.499	0.172	0.770

PPARD +294T > C (rs2016520)	TT	120.0 ± 17.5	75.7 ± 10.1	44.4 ± 12.8	125.8 ± 18.3	79.9 ± 11.1	45.9 ± 13.3
	TC	119.4 ± 16.6	74.6 ± 9.2	44.7 ± 12.1	123.5 ± 14.6	78.4 ± 8.9	45.1 ± 10.7
	CC	120.0 ± 21.3	75.5 ± 9.8	44.5 ± 15.5	125.9 ± 25.7	82.0 ± 17.4	43.9 ± 11.2
	*F*	0.207	1.301	0.088	0.629	0.858	0.703
	*p*	0.813	0.273	0.916	0.533	0.424	0.495

SCARB1 (rs5888)	CC	130.3 ± 19.3	81.2 ± 11.7	49.1 ± 14.7	132.4 ± 19.9	85.1 ± 12.0	47.3 ± 14.2
	CT	131.1 ± 20.6	79.7 ± 11.5	51.4 ± 16.4	132.6 ± 19.1	83.6 ± 11.5	48.9 ± 13.8
	TT	131.7 ± 19.5	82.1 ± 12.6	49.6 ± 11.4	137.6 ± 21.2	78.6 ± 10.3	59.1 ± 19.8↑
	*F*	0.227	2.190	2.615	0.357	2.920	4.830
	*p*	0.797	0.112	0.074	0.700	0.054	0.002 ^c^

SNP, single nucleotide polymorphism; SBP, systolic blood pressure; DBP, diastolic blood pressure; PP, pulse pressure; ABCA-1, ATP-binding cassette transporter A1; ACAT-1, acyl-CoA:cholesterol acyltransferase-1; LDL-R, low density lipoprotein receptor; LIPC, hepatic lipase gene; LIPG, endothelial lipase gene; MTHFR, methylenetetrahydrofolate reductase; MYLIP, the E3 ubiquitin ligase myosin regulatory light chain-interacting protein; PCSK9, proprotein convertase subtilisin-like kexin type 9; PPARD, peroxisome proliferator-activated receptor delta; SCARB1, Scavenger receptor class B type 1. The values of *F* and *p* of the overweight/obese subjects are the interactions between genotypes and overweight/obesity on blood pressure parameters. *P* ^c^ is the *p-*value after permutation correction. ↑, genotype and overweight/obesity interactions to increase blood pressure levels; ↓, genotype and overweight/obesity interactions to decrease blood pressure levels. Sex, age, education level, physical activity, alcohol consumption, cigarette smoking, and hyperlipidemia have been adjusted for the statistical analysis.

**Table 4:** Correlation between genotypes or alleles and blood pressure levels in the normal weight and overweight/obese subjects.

Blood pressure	Genotype/allele	Unstandardized coefficient	Std. error	Standardized coefficient	*t*	*p*
Total population						

SBP	LIPC −250G > A genotype	2.211	0.587	0.081	3.764	0.000
	LIPC −250G > A allele	1.887	0.760	0.054	2.482	0.013
	LIPG 584C > T allele	1.799	0.737	0.053	2.414	0.016

DBP	LIPG 584C > T genotype	1.396	0.403	0.077	3.460	0.001
	LIPG 584C > T allele	1.734	0.466	0.083	3.718	0.000
	PPARD +294T > C allele	−0.964	0.469	−0.046	−2.054	0.040
	SCARB1 rs5888 genotype	−1.298	0.494	−0.062	−2.627	0.009
	SCARB1 rs5888 allele	−1.530	0.564	−0.064	−2.711	0.007

PP	SCARB1 rs5888 genotype	1.945	0.606	0.074	3.208	0.001
	SCARB1 rs5888 allele	2.095	0.694	0.070	3.016	0.003

Overweight/obesity						

SBP	ACAT-1 rs1044925 genotype	2.884	1.277	0.079	2.259	0.024
	ACAT-1 rs1044925 allele	4.816	1.443	0.116	3.338	0.001
	LIPC −250G > A genotype	4.317	0.933	0.154	4.629	0.000
	LIPC −250G > A allele	4.415	1.267	0.117	3.484	0.001
	LIPG 584C > T allele	2.850	1.166	0.085	2.444	0.015

DBP	LIPC −250G > A genotype	1.444	0.579	0.086	2.493	0.013
	LIPC −250G > A allele	1.938	0.782	0.085	2.478	0.013
	LIPG 584C > T genotype	2.167	0.671	0.112	3.232	0.001
	LIPG 584C > T allele	2.783	0.764	0.126	3.644	0.000
	SCARB1 rs5888 genotype	−1.979	0.762	−0.093	−2.597	0.010
	SCARB1 rs5888 allele	−1.788	0.856	−0.075	−2.089	0.037

PP	ACAT-1 rs1044925 allele	2.651	0.941	0.093	2.816	0.005
	LIPC −250G > A genotype	2.862	0.650	0.148	4.405	0.000
	LIPC −250G > A allele	2.414	0.882	0.093	2.736	0.006
	SCARB1 rs5888 genotype	2.989	0.884	0.117	3.383	0.001
	SCARB1 rs5888 allele	2.384	0.994	0.083	2.397	0.017

SBP, systolic blood pressure; DBP, diastolic blood pressure; PP, pulse pressure; ABCA-1, ATP-binding cassette transporter A1; ACAT-1, acyl-CoA:cholesterol acyltransferase-1; LDL-R, low density lipoprotein receptor; LIPC, hepatic lipase gene; LIPG, endothelial lipase gene; MTHFR, methylenetetrahydrofolate reductase; MYLIP, the E3 ubiquitin ligase myosin regulatory light chain-interacting protein; PCSK9, proprotein convertase subtilisin-like kexin type 9; PPARD, peroxisome proliferator-activated receptor delta; SCARB1, Scavenger receptor class B type 1. Sex, age, education level, physical activity, alcohol consumption, cigarette smoking, and hyperlipidemia have been adjusted for the statistical analysis.
